# Management of Thyroid Nodules in Children: A single-center experience

**DOI:** 10.22038/ijorl.2024.78964.3659

**Published:** 2025

**Authors:** Malika El Omri, Oumaima Gabsi, Mouna Bellakhdher, Monia Ghammem, Wassim Kermani, Mohamed Abdelkefi

**Affiliations:** *Department of Ear, Nose, Throat and Head and Neck Surgery, Farhat Hached University Hospital, University of Sousse, Sousse, 4000, Tunisia.*

**Keywords:** Thyroid nodule, Pediatric, Thyroid cancer

## Abstract

**Introduction:**

Thyroid nodules are a common diagnosis in adults. However, in children, they are rare, occurring in only 1-5% of cases. Early diagnosis and prompt management are crucial due to the higher likelihood of malignancy. The aim of this study was to describe the characteristics of thyroid nodules in children and plan their therapeutic management.

**Materials and Methods:**

A retrospective study was conducted on 29 children who underwent surgery for thyroid nodules at our ENT department between 2000 and 2022.

**Results:**

The average age of the patients was 14.4 years, with a clear predominance of females (sex ratio of 0.16). The primary complaint was the appearance of an anterior cervical swelling in 82.7% of cases. The surgical procedures performed on the thyroid gland included isthmectomy in one patient, lobectomy in 16 patients, and total thyroidectomy in 12 patients. Total thyroidectomy was performed in one step in 10 cases and in two steps in 2 cases where papillary carcinoma was found in the final histological examination of the lobectomy parts.

Papillary carcinoma was confirmed in four cases (13.8%) after definitive histological examination. All patients had favourable outcomes. The mean follow-up was 31 months for benign cases and 15 months after the last course of radioactive iodine therapy for malignant cases.

**Conclusion:**

Thyroid nodules are uncommon in children. To evaluate the risk of malignancy in children with thyroid nodules, ultrasound and cytology should be performed. This will help determine the appropriate surgical management.

## Introduction

Thyroid nodules are rare in children, occurring in only 1 to 5% of cases. However, they have a higher potential for malignancy than in adults, with an estimated risk of 22-26% compared to 7-15% in adults. Therefore, it is crucial to promptly diagnose and manage them (1,2). Papillary carcinomas are the most common type of thyroid cancer in children, with a higher incidence of metastases and recurrence than in adults (3,4). The primary treatment for thyroid nodules in children is surgery (5). Despite the possibility of advanced stages, pediatric thyroid cancers have an excellent prognosis (1).

The aim of this study was to describe the characteristics of thyroid nodules in children and plan their therapeutic management.

## Materials and Methods

A retrospective study was conducted on children who underwent thyroid nodule surgery in our ENT department between January 2000 and December 2022. The study included patients up to 18 years of age who were operated on and followed up in our department for thyroid nodules. Patients who were followed up for non-operated thyroid nodules or those who underwent surgery for Graves' disease resistant to medical treatment were excluded.

## Results

Between 2000 and 2022, 4920 patients underwent surgery for thyroid disease over a 22-year period. Out of the total, only 29 patients (0.6%) were children with a mean age of 14.4 years (ranging from 9 to 18 years) and a sex ratio of 0.16. While five patients had a family history of dysthyroidism, no family history of neoplastic pathology was reported. The average duration of symptoms before the first consultation was 11 months (ranging from 1 month to 2 years). Consultation was most commonly sought due to anterior and cervical swelling, as reported by either the patient or their relatives. This was observed in 24 patients (82.7%).

During clinical examination, a goiter was palpated in 86.2% of cases, which developed at the expense of a single lobe. The right lobe was predominantly affected (57%). The nodule had a mean size of 2.5 cm (ranging from 1 to 5 cm). Clinical signs that could predict malignancy, such as hard consistency and irregular boundaries, were found in four patients. Physical examination revealed a lymph node in four patients, with sector III adenopathy palpated in three patients. The adenopathy was firm, painless, and infra-centimetric. In the second case, there were bilateral hard nodes indicating malignancy: a 2 cm mid-jugulo-carotid node and a 1.5 cm subdigastric lymph node. Indirect laryngoscopy was performed preoperatively in 20 cases, which showed two mobile cords, and was not performed in nine cases due to uncooperative patients. Lung auscultation revealed inspiratory crackles in both lungs in one case. 

All patients were found to be biologically euthyroid. Ultrasound scans were performed on all patients, revealing a single thyroid nodule in 17 cases (58.6%) and a multinodular goiter in 12 cases (41.4%). The nodules had a mean size of 28 mm, ranging from 15 mm to 58 mm, with basal nodules being the most common (41.4%). Thyroid nodules were classified according to the European Thyroid Imaging Reporting and Data System (EUTIRADS) as follows: EUTIRADS II in 4 cases (13.8%), EUTIRADS III in 18 cases (62%), EUTIRADS IV in 5 cases (17.2%), and EUTIRADS V in 2 cases (7%) of cases. Cervical lymph node was documented on cervical ultrasound in six patients. One case was suspicious, with bilateral middle and lower jugulo-carotid (sectors III and IV) measuring 3 cm and subdigastric measuring 15 mm (sector IIa).

Fine needle aspiration cytology (FNAC**)** was performed on seven cases of thyroid nodules. It was indicated for nodule classified as EUTIRADS IV with cytology classified as Bethesda II in 4 cases, for nodule classified as EUTIRADS IV with cytology classified as Bethesda III in one case, and nodule classified as EUTIRADS V with cytology classified as Bethesda IV in 2 cases. A FNAC of a lymph node was requested for a patient with suspicious cervical adenopathy. The diagnosis was consistent with metastatic thyroid cancer lymph node.

**Fig 1 (a, b):**
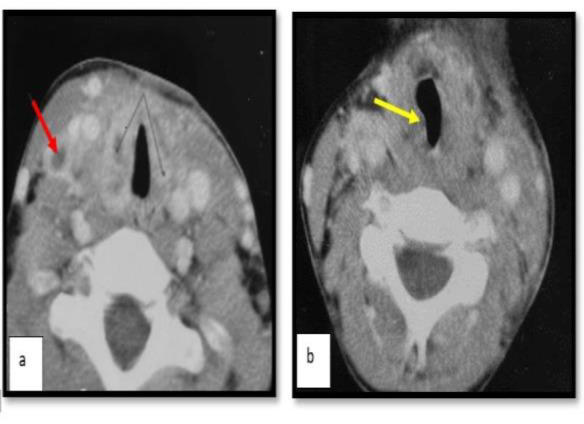
Cervical and thoracic CT scan: suspicious enlargement of the thyroid gland associated with cervical adenopathy and suspicion of laryngeal extension.

**Fig 2 (a,b):**
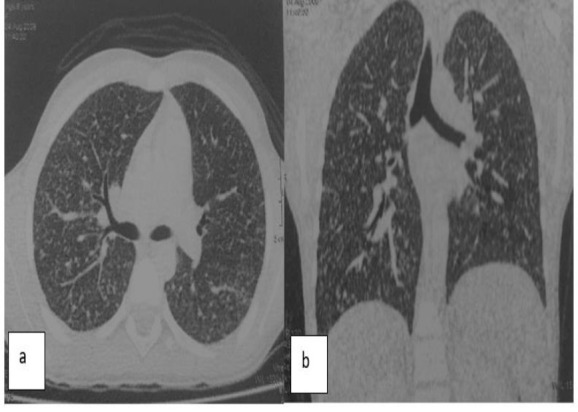
Chest CT scan in axial and coronal sections showing miliary carcinomatosis.

A cervical and thoracic computed tomography (CT) scan was ordered for a patient with a chronic dry cough and cervical lymph nodes. The scan revealed suspicious cervical lymph nodes and a miliary carcinomatosis appearance (Fig. 1,2).

Surgical treatment was necessary for all patients. The indication for surgery was a thyroid nodule associated with signs of compression in 15 cases (51,7%), nodular size > 3 cm in 11 cases (38%), 2 cases classified Bethesda IV (6,9% )and suspicious nodules with metastatic cervical lymph nodes in one case(3,4%) .Ten patients underwent total thyroidectomy (TT) in a single operation, while two patients required two operations due to the presence of differentiated thyroid cancer (DTC) on final histological examination of the lobectomy specimens. Central lymph-node dissection was performed immediately after thyroidectomy (total or partial) in five cases. In three cases, central lymph node dissection was ipsilateral to the suspicious nodule, while in two cases it was bilateral. During the second stage of revision surgery, central dissection was performed on two cases of DTC confirmed by final histological examination. In one patient with clinically and ultrasonographically suspicious bilateral nodes, bilateral lymph node dissection was performed in the lateral sector (sectors II, III and IV) at the same time as the thyroidectomy. The extemporaneous examination of the TT specimen was in favor of DTC. Table 1 summarizes the distribution of patients based on the surgical procedures performed on the thyroid gland and lymph nodes (Table 1).

**Table I:** Number of patients by definitive surgical procedure

Type of surgery	N	%
**TT**	8	27,6
**One stage TT + bilateral central dissection **	1	3,4
**One stage TT + bilateral central and lateral dissection**	1	3,4
**Two- stage TT + contralateral central dissection**	2	7
**Lobectomy** ** + ipsilateral central dissection**	3	10,4
**Lob** **ectomy**	13	44,8
**Isthmectomy**	1	3,4
**Total**	29	100

TT : Total thyroidectomy

The initial stage of surgery for patients with lymph node and lung metastases involved direct laryngoscopy and primary tracheoscopy. No tumour invasion of the larynx or trachea was identified. The final anatomopathological examination revealed benign results in 86.2% of cases, including 11 cases of multinodular goiter, 6 cases of oncocytic adenoma, 4 cases of lymphocytic thyroiditis, and 4 cases of vesicular adenoma. Malignant tumours were found in 4 cases (13.8%). Two patients presented with lateral lymph node involvement. In one case, the involvement was isolated to the central sector, while in the other case, there was involvement of the central and lateral sectors, as well as pulmonary metastases. The characteristics of patients with papillary thyroid carcinoma are summarized in Table 2.

**Table 2:** Summary table of anatomopathological findings in patients with papillary carcinoma

P	Size	N		Multifocality	Bilaterality	Capsular effraction	Extra-thyroid extension	Histologic subtype	TNM
**1**	1.5 cm	1	-		-	-	-	Classic	T1bN0M0
**2**	1.6 cm	1	-		-	-	-	Classic	T1bN0M0
**3**	3 cm	2	+		-	+	-	Vesicular Variant	T2N1aM0
**4**	3.5 cm	4	+		+	+	+	High cell	T4aN1bM1 (lung)

The hospital stays averaged three days, with a range of two to six days. Post-operative courses were straightforward in 15 cases (51.7%) and complicated in 14 cases (48.3%). The most frequent complication was hypoparathyroidism, occurring in 12 patients (41.3%), but it was transient in all cases. Two patients (6.9%) experienced unilateral recurrent paralysis, which was transient and improved well after speech therapy rehabilitation. RAI (Radio Iodine ablation) was indicated in the four patients with DTC. After discontinuing the replacement therapy for 4 weeks, the medication was administered orally. Post-RAI therapy isotope scans revealed mid-cervical fixation of low intensity in 2 patients, moderate intensity in one patient, and intense mid-cervical fixation associated with diffuse bilateral pulmonary fixation in another patient. Cervical ultrasound did not reveal any suspicious lesions. The initial dose administered was 100 mCurie (Ci) in two patients with relatively high postoperative thyroglobulin (Tg) levels and 30 mCi in the other two patients, as shown in Table 3.

**Table 3:** Distribution of patients with papillary carcinoma by stratification of risk of recurrence and dose of radiodine received

P	TNM	Post- operative Tg in stimulation (ng/ml)		Starification of recidivism risk ATA	Number of cures	Total activity (mCi)		Time between 1^st ^cure and surgery (month)
**1**	T1bN0M0	0,1	Low		1		30	7
**2**	T1bN0M0	0,1	Low		2		130	10
**3**	T2N1aM0	13	Intermediate		5		360	8
**4**	T4aN1bM1	685	High		10		1000	9

In benign cases, the average follow-up period was 31 months. Out of the 16 patients who underwent lobectomy, only one presented with a thyroid nodule on the remaining lobe. The nodule was classified as EUTIRADS III and measured 13 mm. It remained stable during 22 months of clinical monitoring.For DTC cases, the mean follow-up period was 15 months after the last I^131^ therapy treatment, and all patients had a favourable outcome. After 10 courses of RAI therapy for the patient, a cervical and thoracic CT scan was ordered, which showed regression of the radiological images. The mean follow-up for benign lesions was 31 months. 

## Discussion

Thyroid nodules are uncommon in children, with a prevalence estimated between 0.5% and 2% (6). Compared to adults, these nodules have a higher malignant potential (7), with a risk of thyroid cancer in children ranging from 9% to 50%, and an average risk of 26%, compared to 5% in adults (5). In our series, the estimated risk was 13.8%.

The prevalence of thyroid nodules increases with age, particularly after puberty (8). Several risk factors have been reported to increase the risk of thyroid cancer in children, including iodine deficiency, previous thyroid disease, exposure to ionizing radiation, and certain genetic predisposition syndromes (9). Additionally, 38% of patients in our series were aged over 15 years. Of the four patients diagnosed with papillary carcinoma, two were over the age of 15. The American Thyroid Association (ATA) states that age may increase the risk of tumour extension and recurrence of thyroid cancer when combined with other factors such as genetic susceptibility and exposure to ionizing radiation (3,10). 

Several studies have investigated the association between autoimmune thyroid disease, specifically Hashimoto's disease, and thyroid cancer (11,12). However, some studies have refuted this theory and found no association between them (13,14). Additionally, numerous studies have confirmed the link between cervical irradiation and the development of thyroid cancer (12). Tucker et al demonstrated that exposure to 20-29 Gray (Gy) is the primary factor in the development of DTC (15), especially in children under 5 years of age (16). In our series, no history of exposure to ionizing radiation was reported.

Currently, genetic predisposition is playing an increasingly important role. Guille discovered that 41% of children with thyroid cancer had a family history of thyroid disease (17). In this series, patients with thyroid cancer did not have a family history of thyroid disease.

For children at high risk of developing thyroid cancer due to genetic syndrome, iodine deficiency, a history of irradiation or thyroid disease, it is recommended that they undergo an annual clinical examination of the cervical area (3). Chiu et al found that a TSH elevation of more than 2.5 mUI/l predicts malignancy in children with thyroid nodules, with papillary carcinoma being the most commonly associated histological variant with an elevated TSH level (18). Routine preoperative calcitonin measurement is not necessary in the pediatric population (3). 

Unlike recommendations for adults, there is currently insufficient evidence in the literature to support the systematic use of ultrasound in high-risk children without abnormalities on clinical examination. Ultrasound is only requested to identify signs of malignancy requiring cytological examination (4). The EUTIRADS classification also applies to children (3). However, unlike in adults, some authors have suggested that an increase in the size of a thyroid nodule in children may indicate malignancy (19). Kotlin (20) suggests that nodules larger than 35mm have a higher risk of malignancy. In our study, only one of the four DTC cases had a nodule larger than 35mm. In the pediatric population, FNAC of nodules does not reduce the number of operations as it does in adults. However, it does encourage more aggressive surgery in the case of suspicious FNAC (21). This procedure is usually performed under sedation or local anesthesia in children (22). According to the ATA, any thyroid nodule in children should undergo FNAC if it increases in size or if there are clinical or radiological signs of malignancy, regardless of its size (3).

The results are expressed according to the Bethesda classification. It is worth noting that the risk of malignancy is higher in children than in adults for each stage, particularly in the Bethesda III stage where the risk is 14.9% in children compared with 7.4% in adults (22). The study included 7 cases where FNAC was performed. The cytology was classified as Bethesda II in 4 cases, Bethesda III in one case, and Bethesda IV in 2 cases. The Bethesda III case involved a DTC on anatomopathological examination. 

The management of thyroid nodules in children based on cytological findings is similar to that in adults, except for Bethesda III stage where surgical treatment is indicated from the outset (9).

The ATA recommends immediate surgery for toxic nodules due to the toxicity of radiodine treatment (3). 

In children with DTC, total thyroidectomy combined with bilateral lymph node dissection of the central compartment and dissection of the affected areas of the lateral compartment are indicated (23, 24, 25). In our study, we performed this surgery on a patient with DTC and metastatic lymph node in the lateral sector. 

Management of medullary thyroid cancer in children is not different from that in adults. However, it is recommended to screen children with a parent carrying the mutation for germline mutation of the RET gene (Rearranged during transfection) at an early stage. If the test is positive, prophylactic thyroidectomy can be performed (3).

Thyroid nodules are mostly benign in children and are dominated by vesicular adenomas (26). The most common histological type in our series was multinodular goiter, with 11 patients.

 It is worth noting that the incidence of thyroid cancer in children is higher than in adults, and the risk of malignancy of nodules varies between 10% and 25%. DTC is the most common endocrine tumour in children, with a prevalence of over 90% (27, 28). Aggressive variants are also more common in children, affecting 15 to 37% of DTCs (26). In children, DTC is often characterized by larger, multifocal and bilateral foci, and a high rate of locoregional metastases at the time of diagnosis (22, 29). Additionally, lung metastases occur in 25% of cases (30). The study identified four cases of DTC, including one case of high cell, one case of vesicular variant, and two cases of classical variant. Two patients exhibited multifocality, bilaterality, and capsular invasion. Lymph node metastases were present in two cases, with one case also exhibiting lung metastases.

Vesicular carcinoma is a rare form of thyroid cancer in children, accounting for less than 10% of cases (31). Unlike DTC, it is less aggressive in this age group (26), and typically presents as a unifocal lesion with few lymph node metastases (3). Medullary carcinoma is estimated to occur in less than 5% of cases in children (26). Our series did not identify any cases of medullary or vesicular carcinoma.

Due to the potential long-term adverse effects, the use of radioactive iodine in the pediatric population is limited (3). Two clear indications exist for the presence of distant metastases (usually pulmonary) and the impossibility of complete surgical removal of the thyroid or lymph nodes (1).

In benign cases, postoperative clinical, biological, and ultrasound monitoring is recommended if a thyroid nodule is present on the remaining lobe when palpated. In a pediatric series of lobectomies for benign nodules published by Akkari in France, the risk of postoperative hypothyroidism was 21%. The risk of new nodules appearing during follow-up was 28%, and the risk of an indication for further thyroid surgery was 5.5% (32).

In cases of DTC, monitoring is based on Tg, anti-Tg antibodies, ultrasound, and whole-body scan Scintiscanning. These tumours have an excellent prognosis, with a 20-year survival rate of over 90% (31). 

The outcome was favourable in all cases in our series. Distant metastases are most often found in the lungs. Unlike adults, children with pulmonary metastases have a low mortality rate due to their maintained avidity to iodine. In such cases, a course of iodine-131 is indicated (33). However, it should be noted that children who have received iodine treatments and are followed up for DTC have a 12% higher risk of developing secondary tumours (34). 

## Conclusion

Thyroid nodules are uncommon in children. To assess the risk of malignancy and optimize management, ultrasound and FNAC are necessary. Multicentric studies with meta-analyses would further enable us to codify therapeutic attitudes in children. 
